# The Use of a Novel NanoLuc -Based Reporter Phage for the Detection of *Escherichia coli* O157:H7

**DOI:** 10.1038/srep33235

**Published:** 2016-09-14

**Authors:** Dandan Zhang, Claudia P. Coronel-Aguilera, Patricia L. Romero, Lynda Perry, Udit Minocha, Carla Rosenfield, Andrew G. Gehring, George C. Paoli, Arun K. Bhunia, Bruce Applegate

**Affiliations:** 1 Department of Food Science, Purdue University, West Lafayette, IN 47907, USA; 2 Department of Biological Science, Purdue University, West Lafayette, IN 47907, USA; 3 Molecular Characterization of Foodborne Pathogens Research Unit, Eastern Regional Research Center, Agricultural Research Service, U.S. Department of Agriculture, Wyndmoor, PA 19038, USA

## Abstract

Rapid detection of the foodborne pathogen *Escherichia coli* O157:H7 is of vital importance for public health worldwide. Among detection methods, reporter phages represent unique and sensitive tools for the detection of *E. coli* O157:H7 from food as they are host-specific and able to differentiate live cells from dead ones. Upon infection, target bacteria become identifiable since reporter genes are expressed from the engineered phage genome. The *E. coli* O157:H7 bacteriophage ΦV10 was modified to express NanoLuc luciferase (Nluc) derived from the deep-sea shrimp *Oplophorus gracilirostris*. Once infected by the ΦV10 reporter phage, *E. coli* O157:H7 produces a strong bioluminescent signal upon addition of commercial luciferin (Nano-Glo^®^). Enrichment assays using *E. coli* O157:H7 grown in LB broth with a reporter phage concentration of 1.76 × 10^2^ pfu ml^−1^ are capable of detecting approximately 5 CFU in 7 hours. Comparable detection was achieved within 9 hours using 9.23 × 10^3^ pfu ml^−1^ of phage in selective culture enrichments of ground beef as a representative food matrix. Therefore we conclude that this NanoLuc reporter phage assay shows promise for detection of *E. coli* O157:H7 from food in a simple, fast and sensitive manner.

The prevalence of Shiga toxin-producing bacterium *Escherichia coli* O157:H7 in the food supply has accounted for hundreds of the reported foodborne outbreaks resulting in thousands of illness in the United States in the past decade^1^,^2^. Outbreak-related food vehicles that are frequently associated with *E. coli* O157:H7 are meat products (especially ground beef)^3^,^4^,^5^,^6^ and fresh produce (such as lettuce, spinach and sprouts)^7^,^8^,^9^. For instance, an outbreak caused by *E. coli* O157:H7 EDL933 in 1982 afflicted approximately 47 people with bloody diarrhea from undercooked meat^10^. In 2006, a large *E. coli* O157:H7 outbreak was linked to contaminated pre-package spinach and 205 individuals were affected across 26 US states with 29% of infected individuals developing severe Hemolytic Uremic Syndrome (HUS)^7^. Rapid and sensitive detection of *E. coli* O157:H7 from food is vital for the prevention of foodborne illness. The extant detection methods for zero-tolerance pathogens of immunological separation and PCR rely on a culture enrichment step. The strength of bacteriophage-based detection methods is that they may exploit this enrichment period to create a reporter signal indicating the presence of the sought-after pathogen. Furthermore, bacteriophages are a useful approach for detection due to their extreme specificity for their hosts and capability of distinguishing between live and dead target cells^11^,^12^.

Bacteriophages have been engineered in the past to include reporter genes so that a fluorescent or bioluminescent signal can be produced only after viable cells are infected and propagated^12^. To date, the reporters used for detection of foodborne pathogens typically include green fluorescent protein (*gfp*), the bacterial luciferase (*luxAB* or *luxCDABE* coupled with *luxI* and *luxR*) and β-galactosidase (*lacZ*)^13^,^14^,^15^,^16^,^17^,^18^,^19^. Recently, a new luciferase, NanoLuc luciferase (Nluc) was engineered from the deep-sea shrimp *Oplophorus gracilirostris* by Promega^20^. Nluc is a small 19 kDa protein and produces bright luminescence with an imidazopyrazinone substrate (furimazine) in a reaction that is independent of ATP. When introduced into group A *Streptococcus*, Nluc, in spite of its eukaryotic origin, has shown a superior sensitivity to firefly luciferase (FFluc) and bacterial luciferase (Lux) in terms of signal strength^21^. Furthermore, it is reported that *E. coli* O157:H7 strains expressing NanoLuc were able to generate a readily detectable signal over two days^22^. While the 6 kb *luxCDABE* cassette is difficult to incorporate into phage genomes, the size of *nluc* gene is small enough (516 base pairs) to have a potential to be inserted into a phage genome with a proper headful packaging^23^. The genetic manipulation of a phage genome using eukaryotic *luc* has so far been limited to the assessment of drug-resistance in the respiratory pathogen *Mycobacterium tuberculosis*^24^.

In this study, NanoLuc for the first time was introduced into the lysogenic bacteriophage ΦV10 for the detection of *E. coli* O157:H7. Previously, Waddell and Poppe^15^ chose transposon mutagenesis to construct a *luxAB*-based ΦV10 reporter phage due to the lack of knowledge of the genetic makeup of this phage. The resultant mutant phage was capable of transducing bioluminescence but failed to propagate on *E. coli* O157:H7 cells. Now the genetic analysis of the ΦV10 phage allows identification of non-essential genes to facilitate the construction of a recombinant phage^25^,^26^,^27^. Therefore in the present report, the previously identified putative gene 37 (*recET*) of phage ΦV10^26^ was replaced by *nluc* via homologous recombination. This novel recombinant phage was used to detect *E. coli* O157:H7 in pure culture and in *E. coli* O157:H7-inoculated ground beef enrichment.

## Results

### Construction of NanoLuc reporter phage

**Figure 1:**
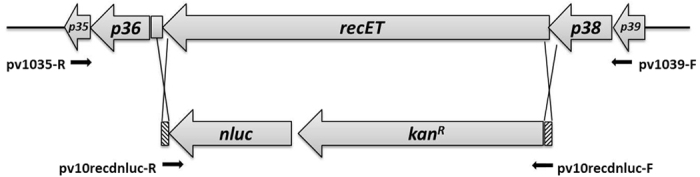
Scheme of the recombination event. The Xs (double crossover) represent the gene exchange sites between the ΦV10 genome and the PCR amplicon containing the *kan*^*R*^*-nluc* cassette amplified by primers pv10recdnluc-F and pv10recdnluc-R. The absence of *recET* and the presence of *kan*^*R*^*-nluc* cassette flanked by the homologous sequences 5′-GTGGCGCCGCGGTGGTGGTTACATAGATGTTTCGTTT-3′ and 5′-TCGCCCGCTGGGATCTGGCGGATCAGCTTGATGGAC-3′ (shown in shaded bars) in the ΦV10 genome was confirmed by sequencing using primers pv1035-R and pv1039-F. The map was drawn based on the sequence of the Enterobacteria phage PhiV10 (GenBank accession NO. DQ126339.2).

The 536 bp DNA fragment encoding the NanoLuc luciferase (*nluc*) along with a translation initiation region was cloned downstream of a constitutive kanamycin resistance determinant. The resultant *kan*^*R*^-*nluc* cassette is approximately 1.7 kb, the size of which is very close to that of the *recET* locus of approximately 1.8 kb. A pair of short homologous sequences flanked by the *kan*^*R*^-*nluc* cassette successfully replaced the complementary regions that are adjacent to *recET*, and the knockout of *recET* was confirmed by sequencing of the DNA fragments around the recombination junctions (Fig. 1). Nluc typically produces a blue light with a maximum emission of 460 nm^20^. Three recombinant isolates emitted blue light when mixed with the Nanoluc-Glo substrate. One isolate B-C2 produced the highest bioluminescence and was chosen to be used in the subsequent experiments. The mutant phage (ΦV10*nluc*) showed spontaneous induction from the B-C2 lysogen. However, when mitomycin C (at a final concentration of 0.5 μg ml^−1^) was added to the culture of strain B-C2 in the exponential phase, the titer of the phage increased by approximately 10-fold. Furthermore the propagation of phage ΦV10*nluc* from the overlay top agar was easily achieved. That phage ΦV10*nluc* was produced from either spontaneously induced lysogens or wild type *E. coli* O157:H7 suggests that the insertion of *nluc* into the phage genome does not compromise phage lytic growth. However, the ΦV10*nluc* plaques appeared to be small pinpointed dimples, as opposed to the large plaques of wild type ΦV10. A similar morphology change was observed in a previous study involving a defective ΦV10 particle^15^. Nonetheless, this defective phage particle was still able to transduce bioluminescence to *E. coli* O157:H7^15^. These observations indicate that the modification of ΦV10 genome can lead to certain phenotypic changes. The visibility of the plaques was increased by the addition of Coomassie Brilliant blue G-250 dye to the top agar (Supplementary Figure S1) without interfering with the phage titer (data not shown).

### Host specificity of bacteriophage ΦV10

Perry *et al*.^25^ previously reported that the O157 antigen was the receptor for ΦV10 as the acetylation of the O antigen by *Oac* during lysogeny. To examine the host specificity, 281 *E. coli* O157:H7 isolates from various sources including: FDA, USDA-ARS and Indiana State Department of Health, were tested for plaque formation with ΦV10. All of the *E. coli* O157:H7 isolates examined formed plaques. Additionally, 39 non-O157:H7 *E. coli* isolates were screened with ΦV10 and no plaques were detected. However 4 of these strains contained the O157 antigen but did not have the H7 antigen. Although this is a limited number of isolates, it suggests specificity for *E. coli* O157:H7.

### Detection of *E. coli* O157:H7 in pure culture by NanoLuc phage

**Figure 2:**
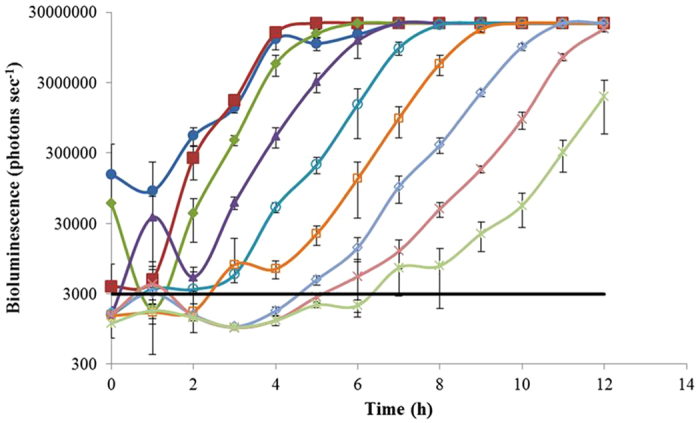
Profile of phage ΦV10*nluc* induced bioluminescence from *E. coli* O157:H7 grown in LB broth. Wild type *E. coli* O157:H7 C7927 was inoculated at 5.40 × 10^8^ (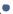), 5.40 × 10^7^ (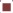), 5.40 × 10^6^ (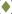), 5.40 × 10^5^ (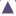), 5.40 × 10^4^ (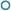), 5.40 × 10^3^ (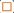), 5.40 × 10^2^ (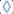), 5.40 × 10^1^ (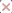), 5.40 × 10^0^ (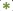) CFU per assay (in a total of 40 ml), and the threshold (—) was set at double the background value. The phage added per assay was 1.76 × 10^2^ pfu ml^−1^. At 1 hour intervals, individual light readings were recorded for triplicate samples. The cutoff of the bioluminescence reading was set up at 2.1 × 10^7^ photons per second which is the upper detection limit of the Sirius luminometer used in this study.

LB broth was used to evaluate the ability of phage ΦV10*nluc* to detect *E. coli* O157:H7. Phage were incubated statically with *E. coli* O157:H7 C7927 cells at dilutions ranging from 5.4 × 10^8^ CFU to 5.4 × 10^0^ CFU in 40 ml broth. Kinetic analysis showed time to detectable luminescence (2x background) increased with decreasing cell populations (Fig. 2). The NanoLuc-induced bioluminescence was readily detected from an initial calculated inoculum of 5.4 cells per assay (in 40 ml) within approximately 7 hours without a pre-incubation period (Table 1). When the initial inoculum was within the range of 10–100 cells per assay, that is 54 cells per assay in this study, it only took approximately 6 hours to detect a positive signal (Table 1).

### Detection of *E. coli* O157:H7 in ground beef enrichment by NanoLuc phage

**Figure 3:**
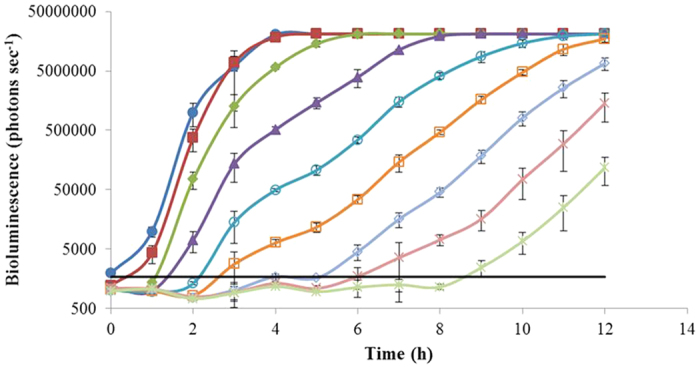
Profile of phage ΦV10*nluc* induced bioluminescence from ground beef artificially contaminated with *E. coli* O157:H7. Wild type *E. coli* O157:H7 C7927 was originally inoculated at 4.68 × 10^8^ (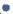), 4.68 × 10^7^ (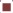), 4.68 × 10^6^ (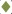), 4.68 × 10^5^ (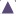), 4.68 × 10^4^ (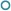), 4.68 × 10^3^ (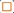), 4.68 × 10^2^ (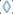), 4.68 × 10^1^ (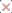), 4.68 × 10^0^ (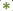) CFU per assay (in a total of 40 ml), and the threshold (—) was set at double the background value. The concentration of phage added was 9.23 × 10^3^ pfu ml^−1^ per assay. At 1 hour intervals, individual light readings were recorded for triplicate samples. The cutoff of the bioluminescence reading was at 2.1 × 10^7^ photons per second which is the upper detection limit of the Sirius luminometer used in this study.

Ground beef has been identified as a major food source that is associated with most recent outbreaks of *E. coli* O157:H7^1^. Therefore ground beef was used as an example to test the ability of our NanoLuc phage (ΦV10*nluc*) for detection of *E. coli* O157:H7 in a complex food matrix during selective enrichment. Ground beef was homogenized in mTSB + n medium and the resultant beef broth was artificially contaminated with *E. coli* O157:H7 C7927 dilutions with a range from 4.68 × 10^8^ CFU to 4.68 × 10^0^ CFU in a total of 40 ml. A similar kinetic response of bioluminescence production was observed from ground beef enrichment as well (Fig. 3). The NanoLuc reporter phage was able to detect *E. coli* O157:H7 C7927 with an initial calculated inoculum as low as 4.68 CFU per assay (in 40 ml) in approximately 9 hours (Table 1). At the level of fewer than 100 cells per assay, this method detects an inoculum of 46.8 cells in a period of approximately 7 hours (Table 1).

## Discussion

Food matrices that are contaminated with Shiga toxin-producing *E. coli* O157:H7 even at the low infectious dose of around 10–100 cells can cause severe outbreaks^28^. A zero-tolerance policy adopted by USDA-FSIS has been used for regulatory-based analysis of samples after *E. coli* O157:H7 was declared as an adulterant in ground beef^2^. In order to detect pathogens at low levels from food, very few of the current detection methods available can bypass enrichment and the subsequent extraction of potential bacteria due to the intrinsic complex nature of food matrices. However, with the use of a bioluminescence reporter phage, the detection signal that is generated during the culture enrichment can be easily non-destructively interrogated in real-time, greatly reducing the time-to-result with minimal labor. In this study, we have constructed a bioluminescent reporter phage from ΦV10. ΦV10 has demonstrated the ability to infect a large number of *E. coli* O157:H7 isolates from a variety of sources and no verified false positives or negatives (with the exception of ΦV10 lysogens) were detected (Supplementary Table S1).

The luciferase gene *nluc* was introduced into ΦV10 genome via the λ Red recombination system using linear PCR amplicons resulting in the reporter phage ΦV10*nluc*. It has been reported that DNA substrates with longer regions of homology have higher recombination efficiency than shorter substrates^29^,^30^. However our attempt to use long homologous substrates was not successful, probably due to low yield from PCR (data not shown). Instead, all the positive recombinant isolates we obtained were generated from short homologous substrates (Table 2). Based on our observation, use of short homologous substrates is adequate to promote the gene exchange in phage ΦV10. While the specific modification of the ΦV10 genome in this study does not affect phage propagation or its ability to transduce bioluminescence to *E. coli* O157:H7, the plaque size was reduced, which made the phage counting difficult. Coomassie Brilliant blue dye is commonly used for protein staining and has been added to agar to detect the fish pathogen *Aeromonas salmonicida*^31^. Here we report adding Coomassie Brilliant blue dye into top agar to improve the visualization of the small plaques with no significant change in titer (data not shown). It may be possible that this staining method can be applicable to other phages as well.

Initial studies to test the ability of phage ΦV10*nluc* to transduce bioluminescence to *E. coli* O157:H7 for detection were conducted using LB broth in a pure culture system. When 1.76 × 10^2^ pfu ml^−1^ of phage ΦV10*nluc* was applied, we detected 5.4 cells per assay (in 40 ml) within 7 hours (Fig. 2). There were luminescent fluctuations at the high inoculation levels (10^8^–10^5^ CFU per assay) in the first two hours. However, such a large load of pathogen in food matrices does not normally occur naturally therefore the analysis of such fluctuations was not included in this report. Ground beef was used as a complex matrix to demonstrate the capability of this phage system to detect *E. coli* O157:H7 in food during selective enrichment. The very low dose of approximately 5 cells per assay (in 40 ml) was detected within 9 hours when using 9.23 × 10^3^ pfu ml^−1^ of phage (Fig. 3). The detection period was further reduced to 7 hours if approximately 50 cells per assay were inoculated. Other phage-based luminescence assays used high concentrations of phage for detection, such as 10^8^ pfu ml^−1 ^16,17, however the phage concentration used in this study was less than 10^4^ pfu ml^−1^, demonstrating the potential to deliver a cost-effective platform which could be easily integrated into current *E. coli* O157:H7 detection regimens. On the other hand, since the developed assay exploits the ability of ΦV10*nluc* to form lysogens, the addition of higher phage concentrations could potentially shorten the time to detection. However it is important to note that ΦV10*nluc* is a temperate phage and upon infection there are two outcomes: either chromosomal integration or lytic propagation. Therefore the initial multiplicity of infection on time to detection needs to be further studied. The resistance marker also allows selective isolation of the resultant lysogens on plates supplemented with kanamycin (Supplementary Figure S2). Isolates can then be screened with luciferin for further verification. The genomic sequence and attachment site of ΦV10 in the host chromosome are known^25^, therefore the ability to trace the origin of the pathogen is not compromised when using sequencing or PFGE for identification.

In summary, a novel NanoLuc-labeled reporter phage was constructed in this study. This recombinant phage was able to stably transduce NanoLuc-induced bioluminescence into *E. coli* O157:H7 to function as a sensitive signaling system. The detection of a very low quantity of *E. coli* O157:H7 (5–6 cells) was achieved in 7–9 hours in pure culture and ground beef enrichment when a small amount of reporter phage (10^2^–10^4^ pfu ml^−1^) was used. In addition, because of the strong signal produced by this NanoLuc luciferase, this reporter phage system could be applied to test other food matrices such as vegetables and dairy products. It is also interesting that the kinetic data plotted in Figs 2 and 3 using this lysogen based method during selective enrichment resemble graphs routinely obtained from using real time PCR for pathogen detection. This suggests the assay could be further developed into a (semi) quantitative method in which time to detection of a specific threshold value could approximate initial levels of contamination. Previously developed *luxCDABE* based reporter phages^17^,^18^ have an advantage over the assay described here as they do not require addition of an exogenous substrate for signal detection. The substrate addition adds complexity to the assay although the brighter signal improves sensitivity. Cost of the reagent has to be considered but at approximately $0.20 per assay in presence/absence format is not prohibitive. In conclusion, this reporter phage can offer a fast and sensitive detection for *E. coli* O157:H7 from food matrices in a simple low cost detection platform.

## Materials and Methods

### Bacterial strains and culture media

Bacterial strains, bacteriophages and plasmids used in this study are listed in Table 3. The *E. coli* O157:H7 C7927 bacterial strain was used for the cultivation of phage ΦV10^32^. Homologous recombination was carried out in a lysogenic strain *E. coli* O157:H7 C7927 (ΦV10) bearing the lambda Red expression plasmid pKD46. For the construction of ΦV10 reporter phage, bacterial strains were grown in Luria-Bertani (LB) broth (Difco Laboratories, MI) or LB agar plates supplemented with antibiotics as needed [ampicillin (Ap), 100 μg ml^−1^; kanamycin (Kan), 50 μg ml^−1^ (Sigma-Aldrich, MO)]. Salt-Optimized Carbon broth medium (SOC) (Clontech Laboratories, Inc, CA) was used for recovery of cells after electroporation. Modified tryptone soya broth (Oxoid Ltd., UK) containing 1% casamino acids (VWR International, PA) with novobiocin (Sigma-Aldrich, MO) of 8 mg l^−1^ (mTSB + n) was used for ground beef enrichment as specified by the USDA-FSIS protocol^33^,^34^. Cell dilutions were done in phosphate buffered saline (PBS) (8 mM Na_2_HPO_4_, 6 mM NaH_2_PO_4,_ 145 mM NaCl, pH7.6). Phage buffer (50 mM Tris, 100 mM MgCl_2_, pH7.6) was used to dilute and preserve the phage stock. LB Top agar (1% (wt/vol) tryptone (Becton Dickinson, NJ), 1% (wt/vol) NaCl (Macron, PA), 0.5% (wt/vol) yeast extract (Hardy Diagnostics, CA) and 0.6% (wt/vol) agar (Alfa Aesar, MA)) was used for the overlay plaque assay.

### Construction of plasmids

The complete coding sequence of the *nluc* gene (GenBank accession no. JQ437370.1) was amplified by touch down PCR using primers nluc-F and nluc-R (Table 2). The NanoLuc luciferase reporter vector pNL1.1 was used as the template. Ready-to-go PCR beads (GE Healthcare, WI) were used as instructed by the manufacturer and the concentration of each primer per reaction was 0.4 μM. The touch down PCR cycling program was performed in two phases^35^. Phase one started with 95 °C for 15 s for initial denaturation, followed by cycles at 94 °C for 15 s per cycle, annealing at successively decreasing temperatures from 68 °C to 56 °C for 15 s (decreasing in increments of 1 °C with 2 cycles per temperature), and then a 72 °C extension step for 1 min per cycle. Phase two consisted of 14 cycles of denaturation at 94 °C for 15 s, annealing at 55 °C for 15 s, and extension at 72 °C for 1 min with a final extension at 72 °C for 2 min. In order to introduce a translation initiation region (Table 2)^36^ upstream of the *nluc* ORF, a second forward primer nluc-F′ paired with nluc-R was used to amplify the nluc-F and nluc-R amplicon. The same PCR cycling program was used as above, except that the annealing temperature of phase one was decreased from 68 °C to 61 °C and in phase two, the annealing temperature was held at 60 °C for 22 cycles. The amplicon consisting of the translation initiation region was cloned into pGEM-T Easy Vector system I (Promega, WI) to create pNluc. A kanamycin-resistance determinant isolated from EZ-Tn5^TM^ < R6Kγori/KAN-2 > Tnp Transposome^TM^ kit (Epicentre Biotechonologies, WI) was inserted into pCR2.1 TOPO vector (Invitrogen, CA) to generate pFSP138. Both pNluc and pFSP138 were digested with *Not*I. Then pFSP138 was further dephosphorylated by shrimp alkaline phosphatase (New England biolab, MA) and ligated with pNluc at the *Not*I site to produce pNluc-Kan.

### Homologous recombination

For constructing a NanoLuc-labeled ΦV10 reporter phage, a gene replacement between *recET* and *nluc* was promoted by using lambda Red recombineering technique for the enterohemorrhagic *E. coli*^29^. Briefly, the forward primer pv10recdnluc-F was designed to contain 36 bases at 5′ end that are homologous to the upstream of *recET* and 21 bases at 3′ end to amplify the kanamycin resistance determinant (Table 2). The reverse primer pv10recdnluc-R was composed of 36 bases at 5′ end to complement the downstream region of *recET* and with 35 bases at 3′ end to amplify the *nluc* sequence (Table 2). The PCR cycling program was 95 °C for 15 s, followed by 38 cycles of 94 °C for 30 s, 60 °C for 30 s and 72 °C for 90 s. DNA prepared from a loopful of a single colony of TOP10 (pNluc-kan) was used as the template. A previously constructed ΦV10 lysogenic strain *E. coli* O157:H7 C7927 (ΦV10) containing the Red expression vector pKD46 was used to promote the gene exchange. The protocol recommended by Sawitzke *et al*.^37^ for making electro-competent cells for lambda Red-driven recombineering was modified accordingly. Briefly, an overnight culture of *E. coli* O157:H7 C7927 (ΦV10) was sub-cultured into 25 ml of LB broth supplemented with ampicillin at 30 °C until the OD_600_ was 0.6. Then L-arabinose solution was added to reach a final concentration of 1 M. After 30 min of incubation at 30 °C with shaking (120 rpm), the cells were made electro-competent at 4 °C. Four microliters of the PCR amplicon digested with *DpnI* was mixed with fresh competent cells and electroporated. These transformed cells were recovered in SOC medium for 3 hours at 37 °C and spread onto LB-Kan agar plates. All the plates were incubated at 37 °C overnight. The positive colonies, which can emit light detected by the Nano-Glo® Luciferase Assay System (Promega, WI), were isolated and grown in LB broth with kanamycin overnight. After centrifugation of the overnight culture at 10,000 g for 10 min, the remaining bacterial cells in the supernatant were removed by a sterile syringe filter with a pore size of 0.2 μm (VWR International, PA). A standard plaque assay^38^ described below was performed to confirm the presence of the presumptive recombinant phage in the culture supernatant. Primers pv1039-F and pv1035-R were designed to amplify either upstream or downstream of the recombination sites. A DNA fragment amplified from a single colony of the presumptive *E. coli* O157:H7 C7927 (ΦV10*nluc*) using primers pv1039-F and pv1035-R was sequenced (the Purdue Genomics Core Facility, Purdue University,West Lafayette, IN) to confirm the deletion of *recET* and the integration of *kan*^*R*^*-nluc* cassette to the ΦV10 phage genome. All the primers used for PCR and sequencing are shown in Table 2.

### Host specificity of bacteriophage ΦV10

Previously obtained *E. coli* strains from Dr. Arun Bhunia’s laboratory collection were screened against bacteriophage ΦV10 by a modified standard plaque assay^38^. Briefly, after LB top agar was melted and tempered to 42 °C in a water bath, 200 μl of the bacteria overnight culture mixed with 100 μl of the appropriate dilution phage was added. The top agar mix was vortexed for two seconds and immediately poured onto the LB base plate to form a top layer. All the plates were incubated at 37 °C overnight after they were solidified.

### Measurement of bioluminescence and propagation of NanoLuc phage

Based on the instructions of the Nano-Glo® Luciferase Assay System, 10 μl of the reconstituted reagent, which was composed of 20 μl of substrate mixed with 1 ml of lysis buffer, was added into a 1 ml aliquot of cell culture or food samples. The light reading was recorded in photons per second by a Sirius luminometer (Berthold Detection Systems GmbH, Germany).

For propagation of this phage, a single colony of the confirmed lysogenic strain *E. coli* O157:H7 C7927 (ΦV10*nluc*) was grown overnight in LB-kan broth at 37 °C with shaking (120 rpm), and then centrifuged to remove cell debris. The supernatant was sterile-filtered to create a crude phage stock. The standard plaque assay described above with a slight modification was performed to enumerate the phage count. Coomassie Brilliant blue G-250 dye (Bio-Rad laboratories, CA) at 1% (wt/vol) in water was added to the original top agar formula to make a final concentration of 0.01% (wt/vol) in the agar medium, to increase visibility of phage plaques.

### Detection of *E. coli* O157:H7 in pure LB broth by using NanoLuc phage

The recombinant phage lysate (ΦV10*nluc*) was purified via dialysis to remove NanoLuc luciferase protein before use for detection. Wild type *E. coli* O157:H7 C7927 was grown in LB broth at 37 °C overnight. Then 10-fold serial dilutions of this stationary phase culture were prepared in sterile PBS. Four ml of each dilution was added to triplicate 36 ml volumes of LB broth where phage ΦV10*nluc* with a final concentration of 1.76 × 10^2^ pfu ml^−1^ was pre-added. LB broth that contained only phage ΦV10*nluc* was included as a background control. All the samples were incubated at 37 °C without agitation. The initial inoculum counts were verified in triplicate by spread plating of the serial-diluted stationary phase culture on LB agar plates. Bioluminescent measurement of a 1 ml sample was taken at 1 hour intervals over a period of 12 hours by using a Sirius luminometer. The detection threshold was set at twice the background value.

### Application of NanoLuc phage to detect *E. coli* O157:H7 in ground beef enrichment

Ground beef was purchased from a local store and processed upon arrival. The USDA-FSIS protocol for preparation of ground beef enrichment broth was followed accordingly^34^. Briefly, one portion of raw ground beef was mixed with 3 portions of modified tryptone soya broth with novobiocin (mTSB + n) in a sterile Seward Ltd. Classic 400 Stomacher strainer bag (Davie, FL) and pummeled for 2 min in a Stomacher LabBlender 400 apparatus (Cooke Laboratory Products, VA). The homogenized beef juice was divided into aliquots of 32 ml in 50 ml sterile polypropylene conical tubes (Corning Inc, NY). An overnight culture of wild type *E. coli* O157:H7 C7927 was diluted as described previously and 4 ml of each dilution in triplicate was added to the tubes containing beef homogenate. Also 4 ml of purified phage lysate was added to each tube to make a final concentration of 9.23 × 10^3^ pfu ml^−1^. Beef homogenate containing only phage lysate was used as a background control. The sampling of culture, the bioluminescence measurement, the incubation time and the set-up of the detection threshold are the same as described previously.

## Additional Information

**How to cite this article**: Zhang, D. *et al*. The Use of a Novel NanoLuc-Based Reporter Phage for the Detection of *Escherichia coli* O157:H7. *Sci. Rep.* **6**, 33235; doi: 10.1038/srep33235 (2016).

## Supplementary Material

Supplementary Information

## Figures and Tables

**Table 1:** Time to detection using the ΦV10*nluc* based assay.

Enrichment medium	Initial inoculum of *E. coli* O157:H7 (CFU/Assay)	Time to detection* (h)
LB broth^a^	5.4 × 10^4^	2.30 ± 0.62
	5.4 × 10^3^	3.07 ± 0.76
	5.4 × 10^2^	4.60 ± 0.10
	5.4 × 10^1^	5.57 ± 0.60
	5.4 × 10^0^	6.70 ± 0.61
Ground beef^b^	4.68 × 10^8^	0.00 ± 0.00
	4.68 × 10^7^	0.50 ± 0.32
	4.68 × 10^6^	1.13 ± 0.12
	4.68 × 10^5^	1.43 ± 0.23
	4.68 × 10^4^	2.23 ± 0.06
	4.68 × 10^3^	2.80 ± 0.30
	4.68 × 10^2^	5.13 ± 0.06
	4.68 × 10^1^	6.47 ± 0.81
	4.68 × 10^0^	8.67 ± 0.29

^*^A positive detection corresponds to 2x background.

^a^Linear regression of initial CFU (log) versus time to detection: y = −0.8781x + 6.6372, R^2^ = 0.9923. The higher initial inocula (5.4 × 10^8^ to 5.4 × 10^5^ CFU/Assay) were excluded due to high variability.

^b^Linear regression of initial CFU (log) versus time to detection: y = −0.8811x + 7.4474, R^2^ = 0.9095.

**Table 2:** The oligonucleotide primers used for this study.

Primer	Nucleotide sequence (5′-3′)
nluc-F	ATGGTCTTCACACTCGAAGATTTCGTTGG
nluc-R	CCGACTCTAGAGTCGCGGCCTTACGCCAGAATGCG
nluc-F′^a^	**ATTAACTTTATAAGGAGGAAAAACAT**ATGGTCTTCACACTCGAAGATTTCG
pv10recdnluc-F^b^	TCGCCCGCTGGGATCTGGCGGATCAGCTTGATGGACCCATCATCGATGAATTGTGTC
pv10recdnluc-R^c^	GTGGCGCCGCGGTGGTGGTTACATAGATGTTTCGTTTCCGACTCTAGAGTCGCGGCCTTACGCCAGAATGCG
pv1039-F	ATGCAGTGGAAAATCATC
pv1035-R	GTCGGCTTAACTTTCTCAC

^a^The sequence of the translation initiation region was shown in bold letters.

^b^The sequence underlined was homologous to the upstream of the kanamycin resistant determinant.

^c^The sequence underlined was homologous to the downstream of the *nluc* gene.

**Table 3:** Bacterial strains, phages and plasmids used and constructed in this study.

Bacteria/Phage/Plasmid	Description	Source or reference
Bacteria		
* E. coli* O157:H7 C7927	Human isolate, host cell for propagation of ΦV10 and its mutant	32
* E. coli* O157:H7 C7927 (ΦV10)	A ΦV10 lysogenic strain	Bruce Applegate
* E. coli* O157:H7 C7927 (ΦV10*nluc*)	A ΦV10*nluc* lysogenic strain	This study
* *OneShot TOP10	General *E. coli* cloning host	Invitrogen
Phage		
* *ΦV10	Non-virulent phage, *E. coli* O157:H7 specific	15
* *ΦV10*nluc*	Δ*recET::kan*^*R*^*nluc* mutant strain of ΦV10	This study
Plasmids		
* *pGEM-T Easy	General cloning vector	Promega
* *pNL1.1	NanoLuc luciferase reporter vector	Promega
* *pNluc	The *nluc* gene with a translation initiation region cloned into pGEM-T Easy vector	This study
* *pFSP138	The kanamycin resistance determinant of Tn5 cloned into pCR 2.1 TOPO vector	Bruce Applegate
* *pNluc-kan	pFSP138 with insertion of kan^R^ determinant upstream of *nluc*	This study
* *pKD46	A lambda Red recombinase plasmid	30
