# Supplementary material for: The Use of a Novel NanoLuc -Based Reporter Phage for the Detection of Escherichia coli O157:H7
Source: Sci Rep. 2016 Sep 14;6:33235. doi: 10.1038/srep33235 (PMC5021930; doi:10.1038/srep33235)
Supplement: Supplementary Information [file srep33235-s1.pdf]

Supplementary Information

for

The Use of a Novel NanoLuc -Based Reporter Phage for the Detection of *Escherichia coli* O157:H7

Dandan Zhang[1], Claudia P Coronel-Aguilera[1], Patricia L Romero[1], Lynda Perry[1], Udit Minocha[1], Carla Rosenfield[2], Andrew G Gehring[3], George C Paoli[3], Arun K Bhunia[1] and Bruce Applegate[1,2]*

[1]Department of Food Science, Purdue University, West Lafayette, IN 47907, USA

[2]Department of Biological Science, Purdue University, West Lafayette, IN 47907, USA

[3]Molecular Characterization of Foodborne Pathogens Research Unit, Eastern Regional Research Center, Agricultural Research Service, U.S. Department of Agriculture, Wyndmoor, PA 19038, USA

*Email: applegate@purdue.edu

Supplementary Table S1.

Bacteriophage ΦV10 specificity assays

Table S1a. *Escherichia coli* O157:H7 strains

Bacterial isolate	Provided by[a]	Plaque formation[b]
E. coli O157:H7 204P	D. Conner	+
E. coli O157:H7 505B	D. Conner	+
E. coli O157:H7 SEA 13A70	J. Johnson	+
E. coli O157:H7 SEA 13A39	J. Johnson	+
E. coli O157:H7 EDL 933	J. Johnson	+
E. coli O157:H7 SEA 13A53	J. Johnson	+
E. coli O157:H7 SEA 13A44	J. Johnson	+
E. coli O157:H7 SEA 13A73	J. Johnson	+
E. coli O157:H7 O1	A. Bhunia	+
E. coli O157:H7 O2	A. Bhunia	+
E. coli O157:H7 O3	A. Bhunia	+
E. coli O157:H7 O4	A. Bhunia	+
E. coli O157:H7 O5	A. Bhunia	+
E. coli O157:H7 O6	A. Bhunia	+
E. coli O157:H7 O7	A. Bhunia	+
E. coli O157:H7 O9	A. Bhunia	+
E. coli O157:H7 O10	A. Bhunia	+
E. coli O157:H7 O11	A. Bhunia	+
E. coli O157:H7 O12	A. Bhunia	+
E. coli O157:H7 O13	A. Bhunia	+
E. coli O157:H7 O14	A. Bhunia	+
E. coli O157:H7 P5	A. Bhunia	+
E. coli O157:H7 P6	A. Bhunia	+
E. coli O157:H7 Q3	A. Bhunia	+
E. coli O157:H7 Q4	A. Bhunia	+
E. coli O157:H7 T3	A. Bhunia	+
E. coli O157:H7 T7	A. Bhunia	+
E. coli O157:H7 T8	A. Bhunia	+
E. coli O157:H7 T10	A. Bhunia	+
E. coli O157:H7 ATCC43890	P. Fratamico	+
E. coli O157:H7 380-94	P. Fratamico	+
E. coli O157:H7 SEA 13B88	P. Fratamico	+

Table S1a-Continued

Bacterial isolate	Provided by[a]	Plaque formation[b]
E. coli O157:H7 G5243	A. Bhunia	+
E. coli O157:H7 G5244	A. Bhunia	+
E. coli O157:H7 G5285	A. Bhunia	+
E. coli O157:H7 G5286	A. Bhunia	+
E. coli O157:H7 G5293	A. Bhunia	+
E. coli O157:H7 G5297	A. Bhunia	+
E. coli O157:H7 G5298	A. Bhunia	+
E. coli O157:H7 G5299	A. Bhunia	+
E. coli O157:H7 G5301	A. Bhunia	+
E. coli O157:H7 G5303	A. Bhunia	+
E. coli O157:H7 G5313	A. Bhunia	+
E. coli O157:H7 G5315	A. Bhunia	+
E. coli O157:H7 G5316	A. Bhunia	+
E. coli O157:H7 557	R. Sanderson	+
E. coli O157:H7 606	R. Sanderson	+
E. coli O157:H7 617	R. Sanderson	+
E. coli O157:H7 704	R. Sanderson	+
E. coli O157:H7 6318	S. Weagant	+
E. coli O157:H7 6319	S. Weagant	+
E. coli O157:H7 6321	S. Weagant	+
E. coli O157:H7 6322	S. Weagant	+
E. coli O157:H7 6323	S. Weagant	+
E. coli O157:H7 6324	S. Weagant	+
E. coli O157:H7 6325	S. Weagant	+
E. coli O157:H7 6326	S. Weagant	+
E. coli O157:H7 6327	S. Weagant	+
E. coli O157:H7 6328	S. Weagant	+
E. coli O157:H7 6329	S. Weagant	+
E. coli O157:H7 6330	S. Weagant	+
E. coli O157:H7 6331	S. Weagant	+
E. coli O157:H7 6332	S. Weagant	+
E. coli O157:H7 6333	S. Weagant	+
E. coli O157:H7 6334	S. Weagant	+
E. coli O157:H7 6335	S. Weagant	+
E. coli O157:H7 6336	S. Weagant	+
E. coli O157:H7 6337	S. Weagant	+
E. coli O157:H7 6338	S. Weagant	+
E. coli O157:H7 6339	S. Weagant	+

Table S1a-Continued

Bacterial isolate	Provided by[a]	Plaque formation[b]
E. coli O157:H7 6341	S. Weagant	+
E. coli O157:H7 6342	S. Weagant	+
E. coli O157:H7 6343	S. Weagant	+
E. coli O157:H7 6344	S. Weagant	+
E. coli O157:H7 6345	S. Weagant	+
E. coli O157:H7 6346	S. Weagant	+
E. coli O157:H7 6347	S. Weagant	+
E. coli O157:H7 6348	S. Weagant	+
E. coli O157:H7 6349	S. Weagant	+
E. coli O157:H7 6350	S. Weagant	+
E. coli O157:H7 6351	S. Weagant	+
E. coli O157:H7 6352	S. Weagant	+
E. coli O157:H7 6353	S. Weagant	+
E. coli O157:H7 6354	S. Weagant	+
E. coli O157:H7 6355	S. Weagant	+
E. coli O157:H7 6356	S. Weagant	+
E. coli O157:H7 6357	S. Weagant	+
E. coli O157:H7 6358	S. Weagant	+
E. coli O157:H7 6359	S. Weagant	+
E. coli O157:H7 6360	S. Weagant	+
E. coli O157:H7 6361	S. Weagant	+
E. coli O157:H7 6362	S. Weagant	+
E. coli O157:H7 6363	S. Weagant	+
E. coli O157:H7 6364	S. Weagant	+
E. coli O157:H7 6380	S. Weagant	+
E. coli O157:H7 6391	S. Weagant	+
E. coli O157:H7 6393	S. Weagant	+
E. coli O157:H7 6394	S. Weagant	+
E. coli O157:H7 6395	S. Weagant	+
E. coli O157:H7 6396	S. Weagant	+
E. coli O157:H7 6397	S. Weagant	+
E. coli O157:H7 6398	S. Weagant	+
E. coli O157:H7 6413	S. Weagant	+
E. coli O157:H7 6414	S. Weagant	+
E. coli O157:H7 6415	S. Weagant	+
E. coli O157:H7 6416	S. Weagant	+
E. coli O157:H7 6417	S. Weagant	+
E. coli O157:H7 6418	S. Weagant	+

Table S1a-Continued

Bacterial isolate	Provided by[a]	Plaque formation[b]
E. coli O157:H7 6447	S. Weagant	+
E. coli O157:H7 6452	S. Weagant	+
E. coli O157:H7 6455	S. Weagant	+
E. coli O157:H7 6456	S. Weagant	+
E. coli O157:H7 6458	S. Weagant	+
E. coli O157:H7 6459	S. Weagant	+
E. coli O157:H7 6460	S. Weagant	+
E. coli O157:H7 6461	S. Weagant	+
E. coli O157:H7 6462	S. Weagant	+
E. coli O157:H7 6463	S. Weagant	+
E. coli O157:H7 6464	S. Weagant	+
E. coli O157:H7 6465	S. Weagant	+
E. coli O157:H7 6466	S. Weagant	+
E. coli O157:H7 6467	S. Weagant	+
E. coli O157:H7 6468	S. Weagant	+
E. coli O157:H7 6469	S. Weagant	+
E. coli O157:H7 6470	S. Weagant	+
E. coli O157:H7 6471	S. Weagant	+
E. coli O157:H7 6472	S. Weagant	+
E. coli O157:H7 6473	S. Weagant	+
E. coli O157:H7 6474	S. Weagant	+
E. coli O157:H7 6475	S. Weagant	+
E. coli O157:H7 6476	S. Weagant	+
E. coli O157:H7 6477	S. Weagant	+
E. coli O157:H7 6478	S. Weagant	+
E. coli O157:H7 6479	S. Weagant	+
E. coli O157:H7 6480	S. Weagant	+
E. coli O157:H7 6481	S. Weagant	+
E. coli O157:H7 6482	S. Weagant	+
E. coli O157:H7 6483	S. Weagant	+
E. coli O157:H7 6484	S. Weagant	+
E. coli O157:H7 6485	S. Weagant	+
E. coli O157:H7 6486	S. Weagant	+
E. coli O157:H7 6487	S. Weagant	+
E. coli O157:H7 6488	S. Weagant	+
E. coli O157:H7 6489	S. Weagant	+
E. coli O157:H7 6490	S. Weagant	+
E. coli O157:H7 6491	S. Weagant	+

Table S1a-Continued

Bacterial isolate	Provided by[a]	Plaque formation[b]
E. coli O157:H7 6492	S. Weagant	+
E. coli O157:H7 6493	S. Weagant	+
E. coli O157:H7 6494	S. Weagant	+
E. coli O157:H7 6495	S. Weagant	+
E. coli O157:H7 6496	S. Weagant	+
E. coli O157:H7 6497	S. Weagant	+
E. coli O157:H7 6498	S. Weagant	+
E. coli O157:H7 6499	S. Weagant	+
E. coli O157:H7 13A17	S. Weagant	+
E. coli O157:H7 13A18	S. Weagant	+
E. coli O157:H7 13A19	S. Weagant	+
E. coli O157:H7 13A21	S. Weagant	+
E. coli O157:H7 13A22	S. Weagant	+
E. coli O157:H7 13A23	S. Weagant	+
E. coli O157:H7 13A24	S. Weagant	+
E. coli O157:H7 13A25	S. Weagant	+
E. coli O157:H7 13A26	S. Weagant	+
E. coli O157:H7 13A27	S. Weagant	+
E. coli O157:H7 13A28	S. Weagant	+
E. coli O157:H7 13A29	S. Weagant	+
E. coli O157:H7 13A30	S. Weagant	+
E. coli O157:H7 13A31	S. Weagant	+
E. coli O157:H7 13A32	S. Weagant	+
E. coli O157:H7 13A33	S. Weagant	+
E. coli O157:H7 13A34	S. Weagant	+
E. coli O157:H7 13A35	S. Weagant	+
E. coli O157:H7 13A36	S. Weagant	+
E. coli O157:H7 13A37	S. Weagant	+
E. coli O157:H7 13A38	S. Weagant	+
E. coli O157:H7 13A39	S. Weagant	+
E. coli O157:H7 13A40	S. Weagant	+
E. coli O157:H7 13A41	S. Weagant	+
E. coli O157:H7 13A42	S. Weagant	+
E. coli O157:H7 13A43	S. Weagant	+
E. coli O157:H7 13A44	S. Weagant	+
E. coli O157:H7 13A45	S. Weagant	+
E. coli O157:H7 13A46	S. Weagant	+
E. coli O157:H7 13A47	S. Weagant	+

Table S1a-Continued

Bacterial isolate	Provided by[a]	Plaque formation[b]
E. coli O157:H7 13A48	S. Weagant	+
E. coli O157:H7 13A49	S. Weagant	+
E. coli O157:H7 13A50	S. Weagant	+
E. coli O157:H7 13A51	S. Weagant	+
E. coli O157:H7 13A52	S. Weagant	+
E. coli O157:H7 13A53	S. Weagant	+
E. coli O157:H7 13A54	S. Weagant	+
E. coli O157:H7 13A55	S. Weagant	+
E. coli O157:H7 13A56	S. Weagant	+
E. coli O157:H7 13A57	S. Weagant	+
E. coli O157:H7 13A58	S. Weagant	+
E. coli O157:H7 13A59	S. Weagant	+
E. coli O157:H7 13A60	S. Weagant	+
E. coli O157:H7 13A61	S. Weagant	+
E. coli O157:H7 13A62	S. Weagant	+
E. coli O157:H7 13A63	S. Weagant	+
E. coli O157:H7 13A64	S. Weagant	+
E. coli O157:H7 13A65	S. Weagant	+
E. coli O157:H7 13A66	S. Weagant	+
E. coli O157:H7 13A69	S. Weagant	+
E. coli O157:H7 13A70	S. Weagant	+
E. coli O157:H7 13A88	S. Weagant	+
E. coli O157:H7 13A90	S. Weagant	+
E. coli O157:H7 13A91	S. Weagant	+
E. coli O157:H7 13A92	S. Weagant	+
E. coli O157:H7 13A93	S. Weagant	+
E. coli O157:H7 13A97	S. Weagant	+
E. coli O157:H7 13A98	S. Weagant	+
E. coli O157:H7 13A99	S. Weagant	+
E. coli O157:H7 13B12	S. Weagant	+
E. coli O157:H7 13B13	S. Weagant	+
E. coli O157:H7 13B14	S. Weagant	+
E. coli O157:H7 13B15	S. Weagant	+
E. coli O157:H7 13B17	S. Weagant	+
E. coli O157:H7 13B18	S. Weagant	+
E. coli O157:H7 13B19	S. Weagant	+
E. coli O157:H7 13B23	S. Weagant	+
E. coli O157:H7 13B25	S. Weagant	+

Table S1a-Continued

Bacterial isolate	Provided by[a]	Plaque formation[b]
E. coli O157:H7 13B26	S. Weagant	+
E. coli O157:H7 13B27	S. Weagant	+
E. coli O157:H7 13B28	S. Weagant	+
E. coli O157:H7 13B30	S. Weagant	+
E. coli O157:H7 13B31	S. Weagant	+
E. coli O157:H7 13B32	S. Weagant	+
E. coli O157:H7 13B33	S. Weagant	+
E. coli O157:H7 13B34	S. Weagant	+
E. coli O157:H7 13B35	S. Weagant	+
E. coli O157:H7 13B53	S. Weagant	+
E. coli O157:H7 13B54	S. Weagant	+
E. coli O157:H7 13B55	S. Weagant	+
E. coli O157:H7 13B56	S. Weagant	+
E. coli O157:H7 13B57	S. Weagant	+
E. coli O157:H7 13B58	S. Weagant	+
E. coli O157:H7 13B59	S. Weagant	+
E. coli O157:H7 13B60	S. Weagant	+
E. coli O157:H7 13B61	S. Weagant	+
E. coli O157:H7 13B62	S. Weagant	+
E. coli O157:H7 13B63	S. Weagant	+
E. coli O157:H7 13B64	S. Weagant	+
E. coli O157:H7 13B65	S. Weagant	+
E. coli O157:H7 13B66	S. Weagant	+
E. coli O157:H7 13B69	S. Weagant	+
E. coli O157:H7 13B70	S. Weagant	+
E. coli O157:H7 13B71	S. Weagant	+
E. coli O157:H7 13B72	S. Weagant	+
E. coli O157:H7 13B73	S. Weagant	+
E. coli O157:H7 13B74	S. Weagant	+
E. coli O157:H7 13B75	S. Weagant	+
E. coli O157:H7 13B76	S. Weagant	+
E. coli O157:H7 13B77	S. Weagant	+
E. coli O157:H7 13B78	S. Weagant	+
E. coli O157:H7 13B79	S. Weagant	+
E. coli O157:H7 13B80	S. Weagant	+
E. coli O157:H7 13B83	S. Weagant	+
E. coli O157:H7 13B84	S. Weagant	+
E. coli O157:H7 13B85	S. Weagant	+

Table S1a-Continued

Bacterial isolate	Provided by[a]	Plaque formation[b]
E. coli O157:H7 13B86	S. Weagant	+
E. coli O157:H7 13B88	S. Weagant	+
E. coli O157:H7 13B89	S. Weagant	+
E. coli O157:H7 13B91	S. Weagant	+
E. coli O157:H7 13B92	S. Weagant	+
E. coli O157:H7 13B93	S. Weagant	+
E. coli O157:H7 13B94	S. Weagant	+
E. coli O157:H7 N009-6-1	NVSL	+
E. coli O157:H7 N045-1-6	NVSL	+
E. coli O157:H7 N192-5-1	NVSL	+
E. coli O157:H7 N317-3-1	NVSL	+
E. coli O157:H7 N336-2-2	NVSL	+
E. coli O157:H7 N336-4-1	NVSL	+
E. coli O157:H7 N396-2-2	NVSL	+
E. coli O157:H7 N404-5-1	NVSL	+
E. coli O157:H7 N931-5-1	NVSL	+
E. coli O157:H7 N1014-7-1	NVSL	+
E. coli O157:H7 N1014-8-1	NVSL	+
E. coli O157:H7 N1014-9-1	NVSL	+
E. coli O157:H7 5906	A. Bhunia	+
E. coli O157:H7 480	A. Bhunia	+

Table S1b. Non-O157:H7 *Escherichia coli* strains

Bacterial isolate	Provided by[a]	Plaque formation[b]
E.coli O157:H⁻ 493-89	P. Feng	-
E. coli O111:H8 HSC 27	R. Johnson	-
E. coli O157:NM MF 7123A	P. Fratamico	-
E. coli O157:NM MF 13180-25	P. Fratamico	-
E. coli O157:H16 P11	D. Thayer	-
E. coli O5:NM 467	B. Blais	-
E. coli O46:H38 HAL EC910004	R. Johnson	-
E. coli O91:H21 HSC 23	R. Johnson	-
E. coli O118:H30 HSC20	R. Johnson	-
E. coli OR:H7 HSC16	R. Johnson	-
E. coli O26:H11 1801-72	P. Fratamico	-
E. coli O26:H11 88.1457	P. Fratamico	-
E. coli O26:H11 2239-69	P. Fratamico	-
E. coli O113:H21 88.0632	P. Fratamico	-
E. coli O22:H8 90.0327	P. Fratamico	-
E. coli O29:NM ATCC 43892	P. Fratamico	-
E. coli O103:H2 87.1368	P. Fratamico	-
E. coli O5:NM 85.0587	P. Fratamico	-
E. coli O55:H7 1880	A. Bhunia	-
E. coli O26:H11 90.0109	A. Bhunia	-
E. coli 103:H11 04.3973	A. Bhunia	-
E. coli 103:H2 04.2446	A. Bhunia	-
E. coli O111:H-IBI-95	A. Bhunia	-
E. coli O111:H11 SH455	A. Bhunia	-
E. coli O145:H8 07865	A. Bhunia	-
E. coli 045:H2 SJ8	A. Bhunia	-
E. coli O121:H19 96.1589	A. Bhunia	-
E. coli O45:H2 SJ7	A. Bhunia	-
E. coli O26:H11 00971	A. Bhunia	-
E. coli O145:H8 07865	A. Bhunia	-
E. coli O26:H11 93-3118	A. Bhunia	-
E. coli O145:NM 03.4699	A. Bhunia	-
E. coli O121:H19 SJ18	A. Bhunia	-
E. coli O26:H11 05-6544	A. Bhunia	-
E. coli O145:NM SJ23	A. Bhunia	-
E. coli O45:H2 96.3285	A. Bhunia	-
E. coli O26:H19 08023	A. Bhunia	-
E. coli O111:NM SJ13	A. Bhunia	-

[a]Bacteria isolates were kindly provided from: D. Conner, Auburn University; J. Johnson, Food and Drug

Administration, Seattle, WA; P. Fratamico, USDA Agricultural Research Service, Eastern Regional Research Center, Wyndmoor, PA; R. Sanderson, Indiana State Department of Health, Indianapolis, IN; S. Weagant, Food and Drug Administration Laboratory, Bothel, WA; National Veterinary State Laboratory, Ames, IA; P. Feng, Food and Drug Administration, College Park, MD; A. Bhunia, Purdue Univeristy; R. Johnson, Health of Animals Laboratory, Canada and B. Blais, Agri-Food Canada.

[b] Phage ΦV10 is specific for *E.coli* O157:H7. All of the *E.coli* O157:H7 isolates formed plaques (+) when infected by phage ΦV10. While no plaques (-) were detected when non-*E.coli* O157:H7 isolates were tested.

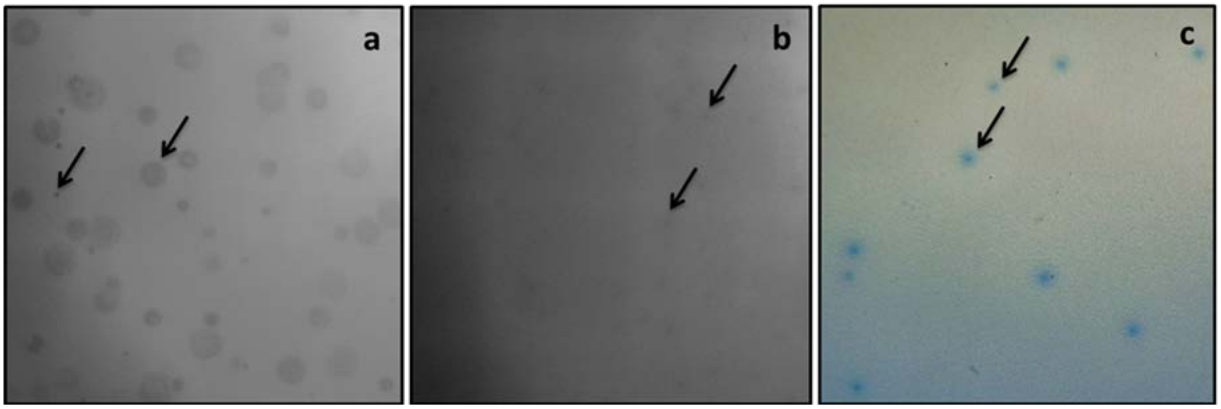

Figure S1. Phenotypic characteristics of phage ΦV10 plaques. (a) Plaques of wild type phage ΦV10 formed in LB top agar; (b) Plaques of phage ΦV10*nluc* detected in LB top agar; (c) Plaques of phage ΦV10*nluc* in LB top agar. The arrows show the representatives of the detected plaques.

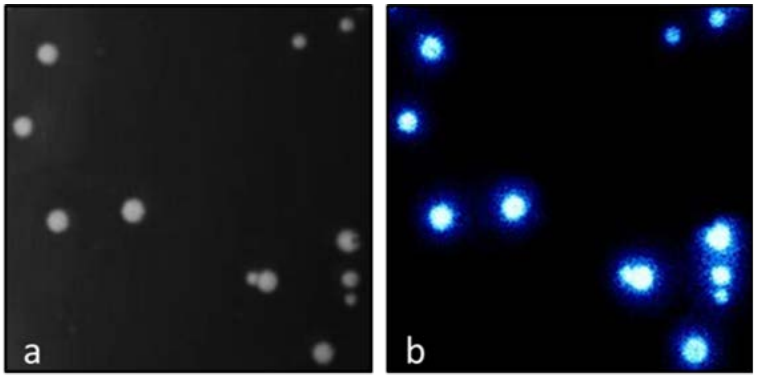

Figure S2. Bioluminsecence emission of phage ΦV10*nluc* lysogens. (a) Lysogens were grown in LB-Kan plates after phage ΦV10*nluc* infected *E.coli* O157:H7 C7927 (ambient light); (b) Bioluminescence emission of lysogens (the same colonies as panel a in dark) after addition of Nanoluc-Glo substrate.
